# Validation of Cardiorespiratory‐Gated Proton Beam Delivery for Stereotactic Arrhythmia Radioablation

**DOI:** 10.1111/jce.70512

**Published:** 2026-09-27

**Authors:** Jagdip Kang, Felicitas J. Bijari, Yanis Chemli, Yen‐Lin Chen, Helen Deng, Nicolas Depauw, Maeva Dhaynaut, Adriano Garonna, Hyeongseok Kim, Marina Macdonald‐Soccorso, Sung‐Hyun Moon, Marc D. Normandin, Rebecca Woodcock, K. Moshe Yaghoubian, Georges El Fakhri, Robert Levine, Kyung‐Wook Jee, H. Immo Lehmann

**Affiliations:** ^1^ Massachusetts General Hospital Boston Massachusetts USA; ^2^ Yale University New Haven Connecticut USA; ^3^ EBAMed SA Geneva Switzerland; ^4^ Massachusetts Institute of Technology Cambridge Massachusetts USA; ^5^ University of Utah Salt Lake City Utah USA

**Keywords:** gating, proof‐of‐concept, radiotherapy, STAR, swine, ventricular tachycardia

## Abstract

**Aims:**

Stereotactic arrhythmia radioablation (STAR) is a promising noninvasive therapy for refractory ventricular tachycardia. Using scanned proton beams for STAR enables precisely focused, high doses of ionizing radiation. Effective mitigation of target motion is critical for safety but remains challenging due to the complex interplay between beam scanning and cardiorespiratory motion. We evaluated the safety and feasibility of accurately delivering gated scanned proton beams to a ventricular myocardial target and compared lesion outcomes of respiratory (single)‐ and cardiorespiratory (dual)‐gated proton beams.

**Methods:**

Six pigs (the first two used as a test of the protocol) underwent baseline CT for treatment planning with a single fractional dose of 35 Gy to a myocardial target in the left ventricular free wall. They were subsequently treated with either single‐ or dual‐gated delivery using scanned proton beams. Treatment response was evaluated by echocardiography, blood analysis and positron emission tomography (PET)‐CT at baseline and 14−15 weeks after irradiation. Lesions were assessed at termination, and tissues were harvested for histopathology.

**Results:**

Average target expansion for intrafractional motion was lower with dual compared to single gating (16%−23% vs. 48%−72%, *N* = 2 in each arm). The irradiated area was distinguishable in vivo on PET‐CT and ex vivo in necropsy at the expected location in all animals. Visual lesion transmurality was confirmed by histology. Lesion sizes were not far from the expected volumes in dual‐gated animals but were larger in single‐gated animals (*p* = 0.3 for between‐group comparison, *N* = 2 in each arm).

**Conclusions:**

The results confirmed the feasibility of the workflow. Dual‐gated irradiation was associated with reduced radiation exposure of the myocardium outside the target volume, and with lesion sizes closer to those planned.

## Graphical abstract

Stereotactic arrhythmia radioablation (STAR) with scanned proton beams was performed in a porcine model targeting the left ventricular free wall (A). Dual gating synchronized proton delivery with both respiratory and cardiac motion was compared to single gating using only respiratory motion (B). Dual gating led to substantially lower CardTV to ITV expansion compared with single gating (C). With dual‐gated irradiation the resulting lesion size was closer to planned sizes (D).

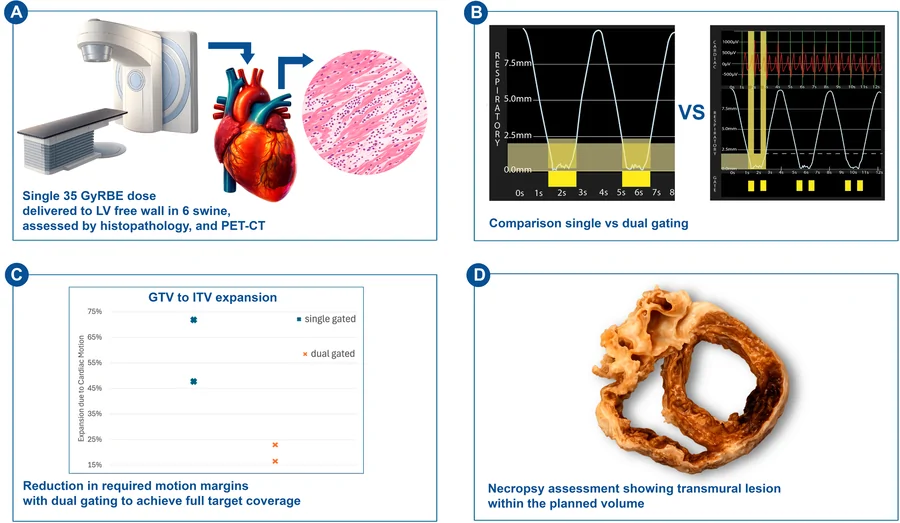

**Abbreviations**
CardTV: cardiac target volume
CT: computer tomography
ECG: single‐lead electrocardiogram
E/e': ratio between early mitral inflow velocity and mitral annular early diastolic velocity
GyRBE: gray radiobiological equivalent
H&E: hematoxylin and eosin
ITV: internal target volume
LV: left ventricle
LVEF: left ventricular ejection fraction
LVFW: left ventricle free wall
PET: positron emission tomography
PTV: planning target volume
STAR: stereotactic arrhythmia radioablation
VT: ventricular tachycardia

## 1 Introduction

Despite recent innovations in catheter ablation, the treatment of refractory ventricular tachycardia (VT) remains a major challenge in cardiac electrophysiology [1, 2]. Stereotactic arrhythmia radioablation (STAR) using X‐ray beams has emerged as a non‐invasive alternative to catheter‐based ablation strategies [3, 4]. STAR targets arrhythmogenic substrates with high‐dose and complex delivered radiation beams. The required dose and underlying biology of STAR remain relatively unknown. Interest is growing in using particle beams such as proton beams as an alternative radiation modality. These provide improved spatial dose distribution, and offer the potential for delivery of high radiation doses with lack of exit dose, sparing non‐involved myocardium, cardiac sub‐structures, and organs‐at‐risk (OAR) from radiation [5].

Effective mitigation of target motion is critical for cardiac protection and optimized radiation dose distribution. Traditionally, conventional respiratory motion mitigation strategies have been employed, including abdominal compression, internal margins, and/or respiratory gating [6]. However, in spite of the importance of integrating both respiratory and cardiac motion in future motion management strategies [7], cardiac motion remains a unique challenge due to its complexity and rapid cyclic nature. To date, cardiorespiratory motion is most often covered using additional margins that encompass possible target positions along the respiratory and cardiac cycles [8]. This leads to excessive irradiation of the surrounding healthy myocardium with the target doses [9] and potentially leads to significant downstream side effects.

CardioKit (EBAMed SA, Geneva Switzerland) is an investigational, hands‐free, cardiorespiratory image‐guidance system for target monitoring and cardiorespiratory gated irradiation. It combines automated ultrasonographic image acquisition with an artificial intelligence (AI)–driven algorithm capable of real‐time cardiac motion monitoring during radiotherapy [10]. Simulation studies under different gating scenarios have shown the potential for reduced irradiation volumes when using combined cardiorespiratory gating [11].

Here, we present the results of a preclinical end‐to‐end study with a clinically relevant workflow comparing lesion outcomes between animals that underwent proton STAR with cardiorespiratory gating using CardioKit with those treated using respiratory gating alone, at 14−15 weeks follow‐up.

## 2 Methods

### 2.1 Study Design

The large‐animal study protocol was reviewed and approved by the Massachusetts General Hospital Institutional Animal Care and Use Committee (IACUC). Six healthy male Yucatan pigs were included in this study. Three underwent single‐gated proton STAR and three underwent dual‐gated proton STAR. The first two animals were treated and analyzed as a test of the protocol before treating and analyzing the subsequent four animals. All animals underwent baseline pre‐treatment computed tomography (CT) imaging for radiation therapy planning and positron emission tomography–computed tomography (PET‐CT) imaging, as an in vivo method to assess accurate dose delivery. A radiation dose of 35 Gray radiobiological equivalent (GyRBE) was used, a level which has been associated with the formation of clear transmural lesions in the left ventricle in other animal studies using MRI and visual lesion assessment [12, 13]. After radioablation, animals were followed up to 14−15 weeks, undergoing multiple post‐irradiation PET‐CTs (three for the first two test animals and two for the following four). The time points for post‐irradiation imaging were chosen based on previously described time frames for observations of myocardial changes after proton beam ablation [13, 14]. At all study time points, transthoracic echocardiography was performed to evaluate cardiac function, and blood samples were collected. At the time of termination, animals were euthanized, and necropsy and gross pathology assessments were performed. There was no randomization. Analyzing pathologists were blinded to the identity of the respective samples and no other blinding was used.

### 2.2 Pre‐and Post‐Treatment Imaging

All animals underwent echocardiographic imaging twice before and twice after radioablation. LV ejection fraction (EF) was assessed using the Simpson method and global longitudinal strain. E/e' was measured to assess diastolic function.

As an in‐vivo control of successful irradiation of the target area, serial [^18^F](4‐Fluorophenyl) triphenylphosphonium ([^18^F]‐FTPP^+^) PET‐CT imaging was performed to track longitudinal changes in cardiac tissue membrane potential (ΔΨ_T_). Prior to PET imaging, an attenuation‐correction CT scan was acquired. The image acquisition, reconstruction, and analysis methods were developed in our laboratory [15, 16, 17]. Following transformation of the ΔΨ_T_ map into short axis, average segmental ΔΨ_T_ values were computed for the 17‐segment American Heart Association model and were compared between treated and control segments at baseline and follow‐up timepoints using a Wilcoxon signed rank test. The “treated” segments were determined as segments that were largely included in the planning target volume (PTV). Segments outside of the targeted area (i.e., on the interventricular septum) were used as a control. The analysis was not blinded.

Following baseline PET, simulation CTs were performed on the same scanner for radiotherapy planning. During imaging, the animals were breath‐held at the end of exhale for pre‐contrast and contrast ECG‐gated dynamic cardiac CTs. To facilitate the modeling of the cardiac motion, cardiac CTs were reconstructed retrospectively for 20 cardiac bins (i.e., from 0% time of the R−R interval to 95% R−R) with a slice thickness of 1.25 mm and a pixel size of ~0.98 mm × 0.98 mm. The simulation CTs included the immobilization device, the whole animal cross‐section laterally, and the entire lung longitudinally.

### 2.3 Treatment Planning and Proton Therapy

The location and size of the targets were determined by a radiation oncologist and a cardiac electrophysiologist on the diastolic phase of the contrast cardiac CTs (i.e., 80% R−R). The target was centered on the left ventricular‐free wall (LVFW) to guarantee a reproducible target location, encompassing the papillary muscle and the full myocardial thickness with a circular shape of 2.5 cm diameter (3.5 cm diameter for the first two test animals). This cardiac target volume (CardTV) was expanded by internal margins to account for cardiac motion based on the treatment techniques (i.e., dual gating vs. single gating), resulting in the internal target volume (ITV). This was performed automatically by deformable registration (on all phases of the cardiac CTs for single‐gated animals and gating phases for dual‐gated animals) and later checked visually by a clinician. The dual‐gated treatment (animals 1, 3, and 5) employed a cardiac gating window covering only the diastolic phases (i.e., 70% R−R to 100% R−R), whereas a larger internal margin accounting for all cardiac phases (i.e., 0% R−R to 100% R−R) was added to the CardTV for single‐gated treatment (animals 2, 4, and 6). This is schematically illustrated in Figure 1. An additional treatment margin of 5 mm was added isotropically to the ITV, creating the PTV. This margin accounts for setup uncertainty and the residual motion from the 2 mm respiratory gating window. Likewise, all OARs were initially contoured on the 80% R−R contrast CT and expanded by the corresponding cardiac motions. All contours were transferred to the corresponding average pre‐contrast CTs for radiotherapy planning.

**Figure 1:**
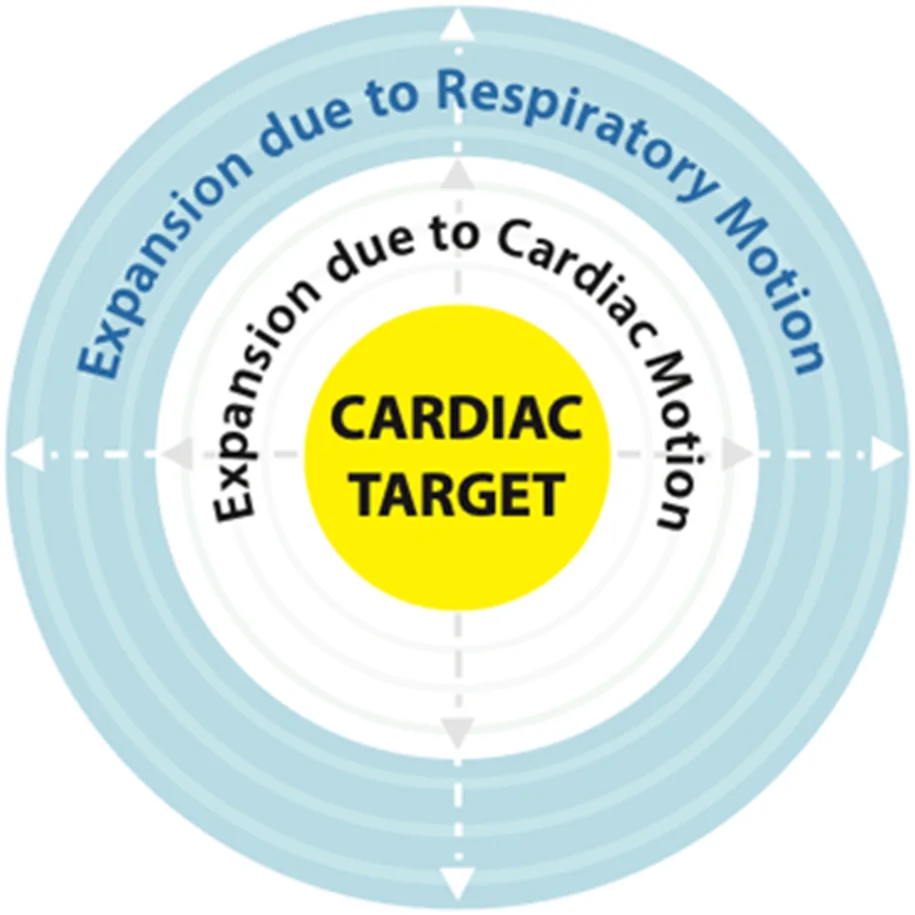
Diagram illustrating the difference in target size between no gating (outermost circle), single gating (central circle), and dual gating (innermost circle). With dual gating, the treatment target corresponds most closely to the cardiac target delineated on a static CT. With single gating, the target size is enlarged to encompass all possible target positions during the cardiac cycle. Without any gating, the target size is further enlarged to encompass all possible target positions during the respiratory cycle. The diagram is highly simplified for clarity; in reality, targets and expansions may not be circular and/or uniform.

The Astroid treatment planning system (decimal LLC, Sanford, FL, USA) was used to design, optimize, and simulate STAR. Treatment plans were generated using two fields, delivering a 35 GyRBE PTV dose in a single session, as illustrated in Figure 2. The target coverage was optimized while enforcing clinically relevant dose limits for the left anterior descending artery (LAD). Depending on their position, parts of the mitral valve and left circumflex artery were included in the PTV for some animals. In the absence of cardiac gating (i.e., in the single‐gated treatment), the four‐times layer‐repainting delivery technique was used to reduce the interplay effects caused by the cardiac motion [18].

**Figure 2:**
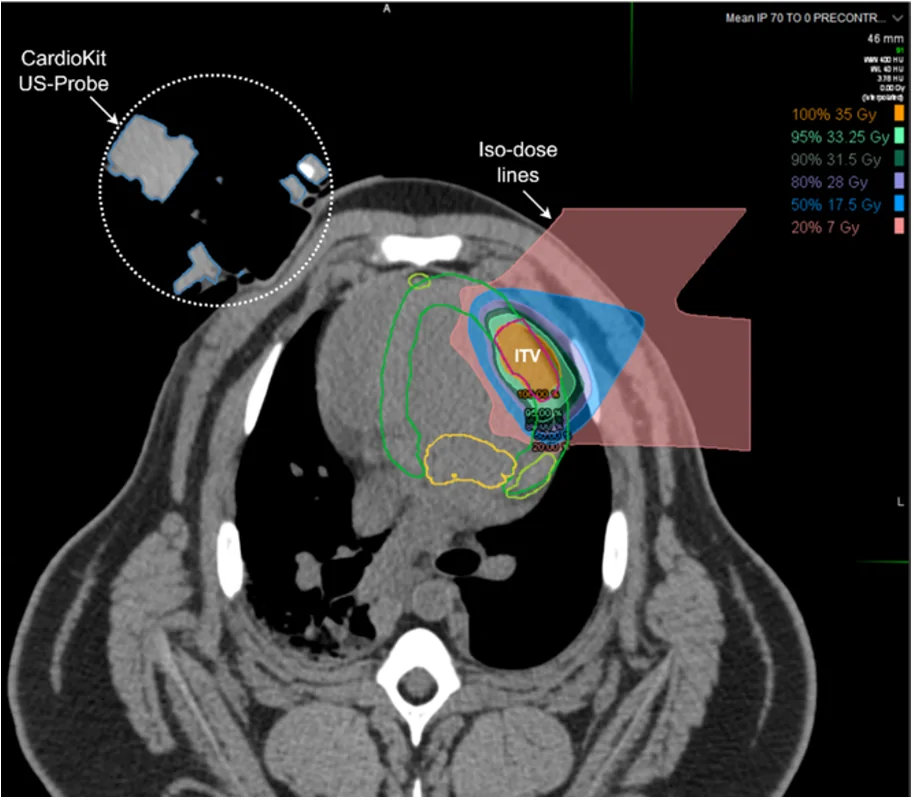
Illustration of the two treatment beams (color wash) and high‐dose target contour (ITV in red) on the LVFW for one example animal (dual‐gated treatment). Irradiated volumes receiving 100%, 95%, 90%, 80%, 50%, and 20% of Rx dose (35 GyRBE) are shown by the corresponding iso‐dose lines (IDLs). The lowest isodose line shows the beam path in air. Contours of the left ventricle, mitral valve, and coronary arteries are shown in green, yellow, and light green, respectively.

For irradiation, the IBA (Ion Beam Applications SA, Louvain‐La‐Neuve, Belgium) proton system at the Massachusetts General Hospital Francis H. Burr Center was used. A gantry beamline with Pencil Beam Scanning modality was used for optimal beam configurations. The treatment setup was aided by the two orthogonal, gantry‐mounted 2D X‐ray imaging systems.

### 2.4 CardioKit System

The CardioKit system records both real‐time soft tissue ultrasound images and surface ECG signals (lead I, II, or III), to process the data required for single‐ or dual‐gated beam delivery. Hands‐free parasternal transthoracic echocardiography with a compact probe was performed at baseline [10]. The system relies on a convolutional neural network to calculate, in real‐time, the extent of ultrasound image displacement due to breathing to a reference image acquired in the same phase of the cardiac cycle. The supervised training of the AI regression model was based on human and porcine data. The respiratory gating computation is based on ultrasound imaging, whereas cardiac gating relies on automatic detection of ECG R‐peaks [11]. The conditions to resume/pause gated irradiation are checked every 1 ms. Response times of the proton beam delivery system to gating commands (i.e., latency) were characterized prior to treatment and automatically compensated to enable beam delivery only when the heart was within the exact cardiorespiratory window. Internal delay time for signal and image processing are also accounted for to make sure the beam is delivered within the intended gating window [19]. The dual gating method in CardioKit is illustrated in Figure 3. The functional testing of the system (as well as the novel scanning controller) for this study will be described in a separate publication.

**Figure 3:**
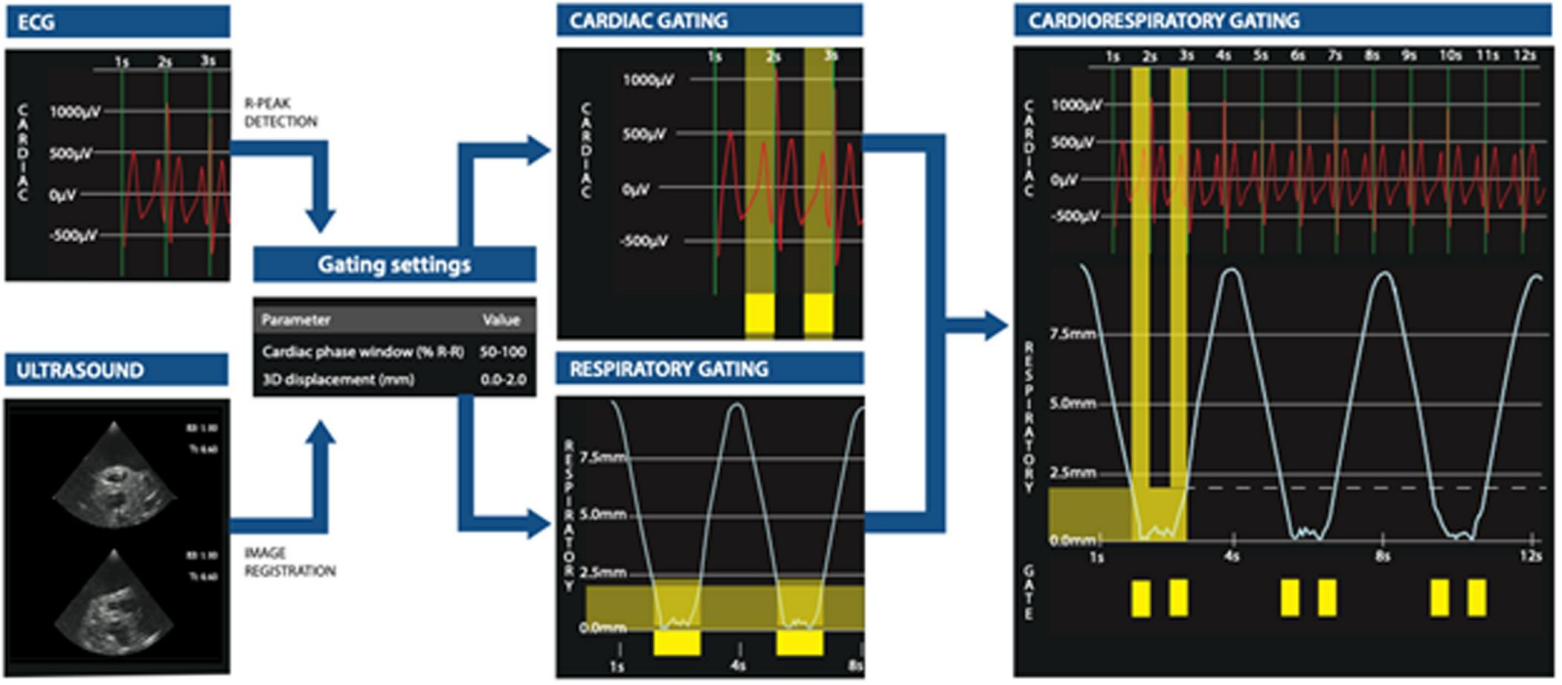
Diagram illustrating the dual gating method using the CardioKit. A single‐lead surface ECG signal is collected, and the R‐peaks of the QRS complex are automatically detected (top‐left). In parallel, bi‐plane ultrasound images from a compact probe are acquired and binned using the ECG data (bottom‐left). The images are registered to determine in real‐time the target respiratory motion. The dual‐gating parameters entered by the user in the graphical user interface are used to calculate the timing of gating signals for cyclically activating and stopping beam delivery according to the patient's cardiorespiratory signals (center). The information on cardiac and respiratory motion is combined to generate gating signals to the therapy machine to deliver the beam only when the target is in the desired location (right).

### 2.5 Blood, Tissue Sampling, and Lesion Quantification

At each study time point, blood samples were collected and analyzed for blood cell composition, red blood cell parameters, and liver/kidney function parameters. At the completion of the study, the animals were sacrificed, and a necropsy was performed. Adjacent organs in the upper abdomen, including the liver, spleen, and diaphragm, were inspected for visual changes possibly related to the radiation treatment. Pericardial samples were collected from the irradiated area for histological analysis, and from the right‐sided pericardium as a control. After macroscopic lesion size measurement and photographic documentation (performed by a trained cardiac surgeon), the heart was cut into multiple 5‐mm‐thick transversal sections to verify the transmurality of the lesion. Samples for histology were taken from the center and the margin of the radiation lesion, and from the non‐irradiated myocardium as a control. All histological samples were stained with hematoxylin and eosin (H&E) and trichrome. The measurements of lateral lesion size and tissue thickness were combined to calculate the lesion volume, modeling its shape as an ellipsoid (Figure 7). The measured volumes of the macroscopic lesions were compared to the estimated volumes of the irradiated LVFW for different dose levels in % of the prescription target dose at the time of termination (IDV_%). In the estimation, the physiological growth of the heart from baseline to termination was taken into account using the motion‐informed image‐derivative‐based deformable image registration method [20].

### 2.6 Statistical Analysis

Statistical significance was assessed using the Mann−Whitney *U* test for all variables, given the lack of a normal distribution. For PET‐CT analysis, the Wilcoxon signed‐rank test was used. *p* < 0.05 were considered statistically significant. Statistical analyses were performed using R Statistical Software (v4.3.0; R Core Team 2023).

## 3 Results

### 3.1 Feasibility and Safety of Dual‐Gated Workflow

All animals underwent the designated treatment strategy, confirming the feasibility of the planned workflow. The first two animals were treated and analyzed as a test of the protocol. Beam delivery times for both beam angles were 4−5 min for single gating (*N* = 2) and 9−17 min for dual gating (*N* = 2). The CardTV to ITV expansion observed with dual gating was less than half that observed with single gating (16%−23% vs. 48%−72%, *N* = 2 in each arm) (Figure 4). CardTV ranged from 5.6 to 6 mL for animals 3−5 but was 3.7 mL for animal 6. The single‐gated animal 4 showed the largest PTV, whereas animal 6 (single‐gated) showed a PTV comparable to the dual‐gated animals, due to larger target expansion. There were no significant differences in the maximum doses (D_1%_) delivered to the same OARs between the dual‐gated (*N* = 2) and single‐gated (*N* = 2) animals: 11.8 ± 0.7 versus 11.9 ± 0.4 GyRBE (*p* = 0.9) for the LAD, 19.0 ± 21.3 versus 24.2 ± 13.8 GyRBE (*p* = 0.8) for the left circumflex artery, and 7.7 ± 8.8 versus 21.7 ± 4.2 GyRBE (*p* = 0.2) for the mitral valve.

**Figure 4:**
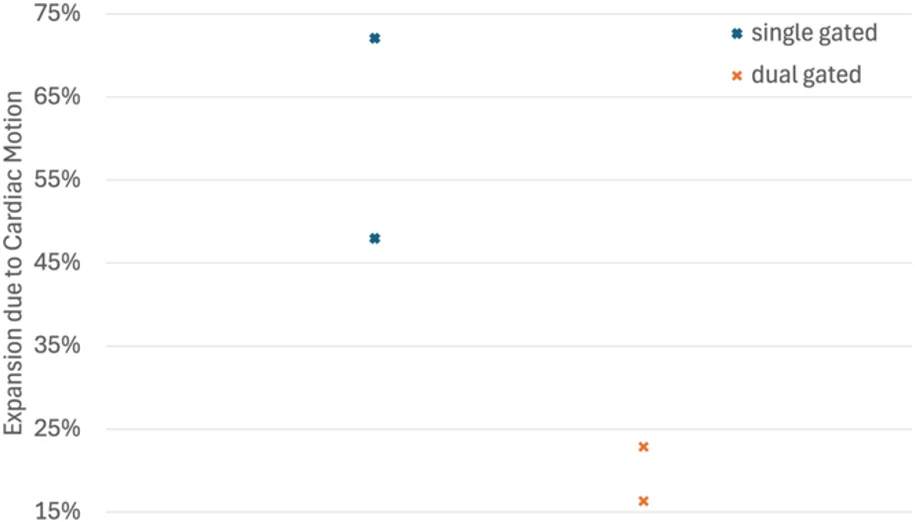
Cardiac target volume (CardTV) to internal target volume (ITV) expansion for single‐gating (*n* = 2) in blue and dual‐gating (*n* = 2) in orange.

This finding was expected, as the treatment strategy prioritizes adherence to the LAD dose constraint over target coverage, with the LAD considered a dose‐limiting organ. The maximum dose to the LAD was 11.8 ± 0.5 GyRBE over the full cohort. In contrast, the doses to the left circumflex artery and mitral valve were relatively high (21.6 ± 14.9 and 14.7 ± 9.9 GyRBE, respectively) in both gating modalities because these structures were intentionally included within the PTV margin.

Delivery of radiation did not have any significant side effects in any of the subjects. The animals did not show any signs of pain or discomfort and did not require any special treatment. All animals developed an expected (from proton therapy oncological clinical practice) skin discoloration as a procedure‐related adverse event, which did not require treatment. The blood analysis was mostly unremarkable and showed only signs of mild anemia at all time points. There were no pathological findings regarding liver and kidney function.

Echocardiographic measurements of LV systolic and diastolic function are summarized in Table 1. There was no statistically significant difference from baseline to end‐of‐study in LVEF, LV global longitudinal strain, or diastolic cardiac function, nor were there any statistically significant differences in these parameters between the two gating types. No structural valve changes were observed.

**Table 1:** Echocardiographic results.

		Protocol test		Analysis			
	Animal identifier	1	2	3	4	5	6
	Gating type	Dual	Single	Dual	Single	Dual	Single
LVEF [%]	Baseline	58.6	62.2	69.7	62.3	66.5	54.3
	End of study	73.0	60.3	65.4	56.5	67.5	61.8
LV global longitudinal strain [%]	Baseline	−26.1	−17.5	−27.9	−22.8	−30.7	−29.2
	End of study	−37.0	−22.3	−28	−23.5	−20.9	−32.6
Diastolic function E/e'	Baseline	1.4	13.1	7.0	3.9	5.8	4.9
	End of study	2.0	7.8	9.8	9.3	6.9	10.3

### 3.2 Lesion Assessment

All animals completed the follow‐up period without any complications. PET‐CT was used for lesion assessment in vivo during follow‐up. At 14 weeks post‐treatment, ΔΨ_T_ was numerically less negative in treated compared to control segments, a sign of impaired cardiac tissue function. The difference in average ΔΨ_T_ between treated and control segments assessed at the end of the study (14 weeks) was 22.4 mV (dual‐gated animal 3), 44.4 mV (single‐gated animal 4), and 47.0 mV (single‐gated animal 6). The extent of ΔΨ_T_ difference was lower for segments outside but near the target, as expected: 10.4 mV (dual‐gated animal 3), 15.0 mV (single‐gated animal 4), and 23.5 mV (single‐gated animal 6). A graphical representation of a representative animal (animal 3, dual‐gated) is provided in Figure 5. The full report of the membrane potential PET‐CT results will be provided in a separate image assessment paper.

**Figure 5:**
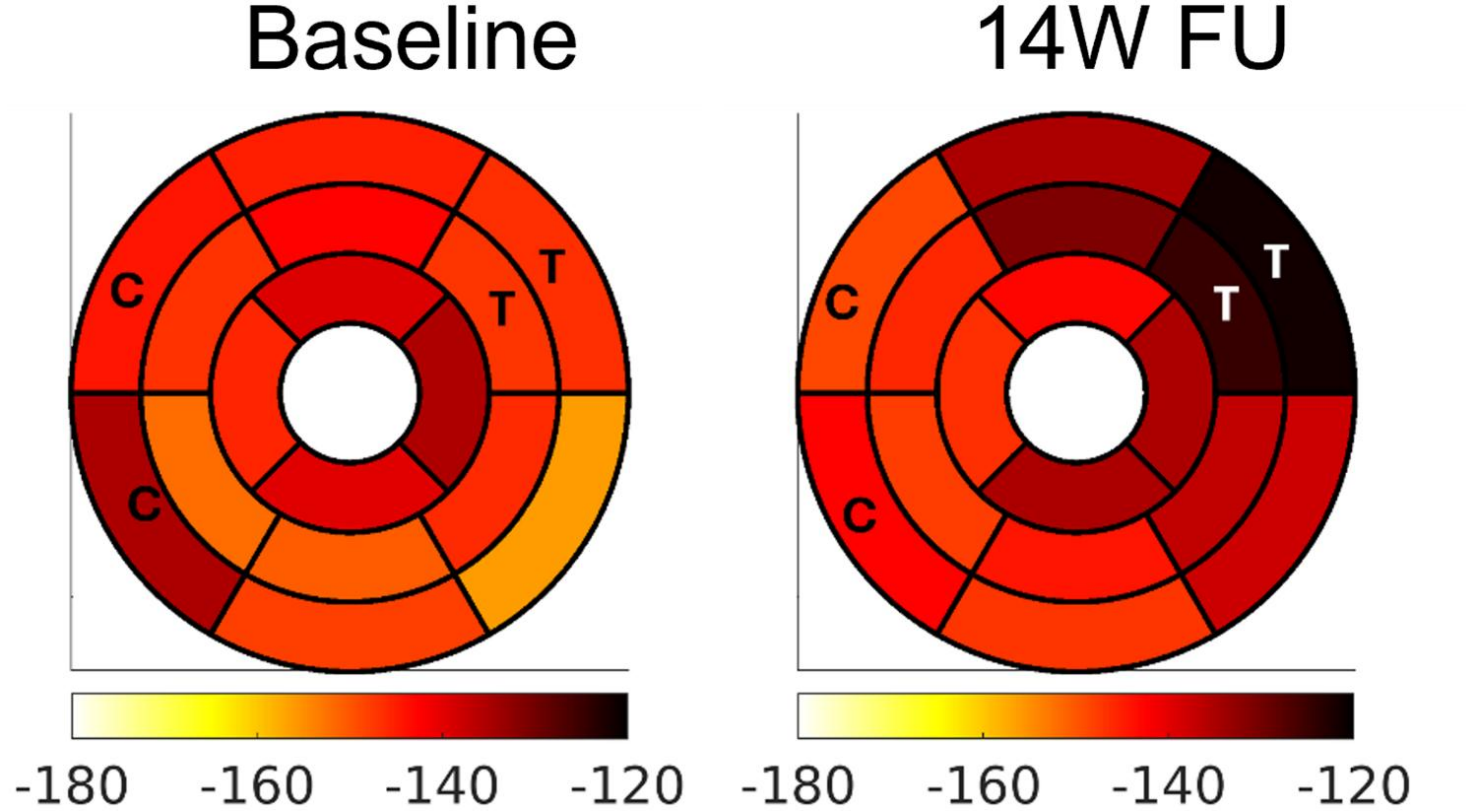
Polar maps of ΔΨ_T_ at baseline and 14‐week follow‐up for the dual‐gated animal 3. “T” indicates treated segments (included in the PTV), “C” indicates control segments.

For all animals, the lesion visually observed at necropsy was at the planned location (i.e., consistent with the treatment plan) and was confirmed by histopathology. Dissection of the lesion revealed macroscopic fibrosis (Figures 6 and 7). In animals 3−6, the lesions also affected the myocardium, as planned. For animal 4, a thickening of the medial part of the left upper lung lobe was visible. These observed changes in the lung tissue were consistent with the inclusion of the upper left lobe in the targeted radiation zone.

**Figure 6:**
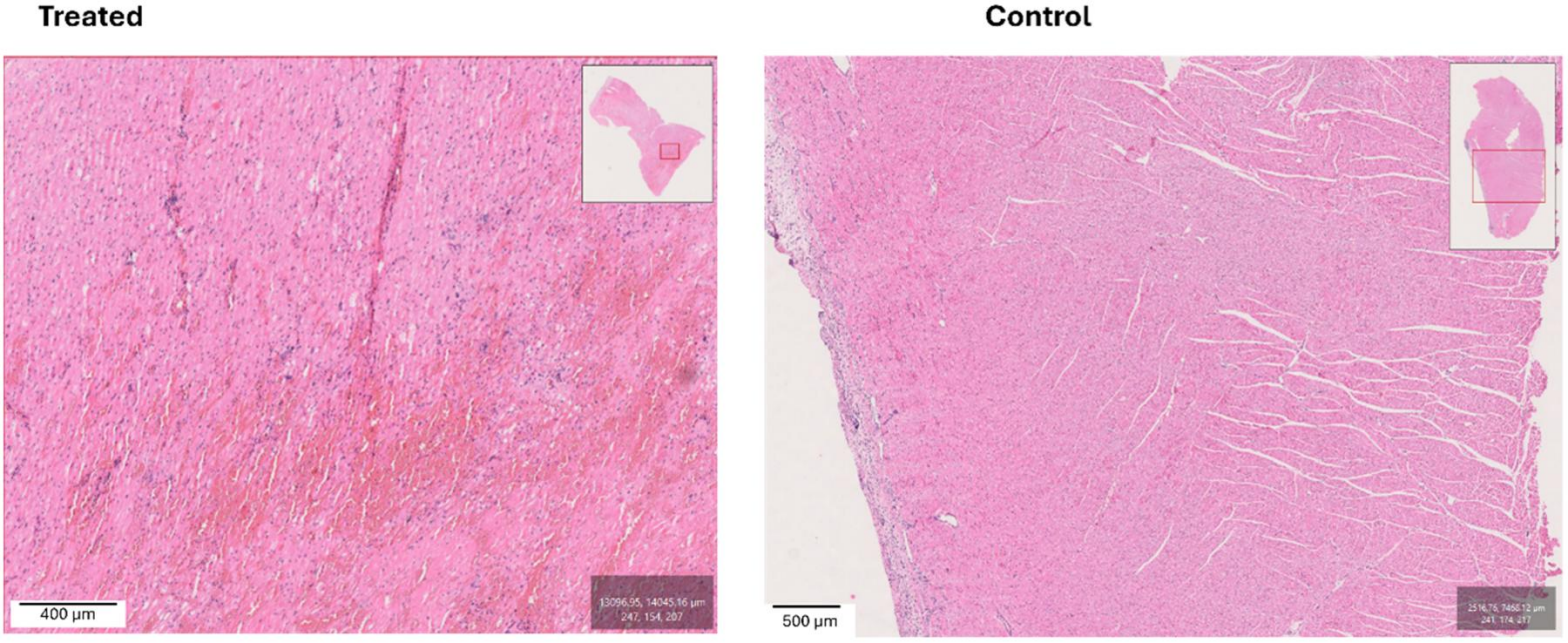
Histopathological samples (H&E stain) of treated (left) and control (right) myocardium in one example animal, showing extensive hemorrhage and necrosis with thromboses in the treated area and unremarkable myocardium in the control tissue.

**Figure 7:**
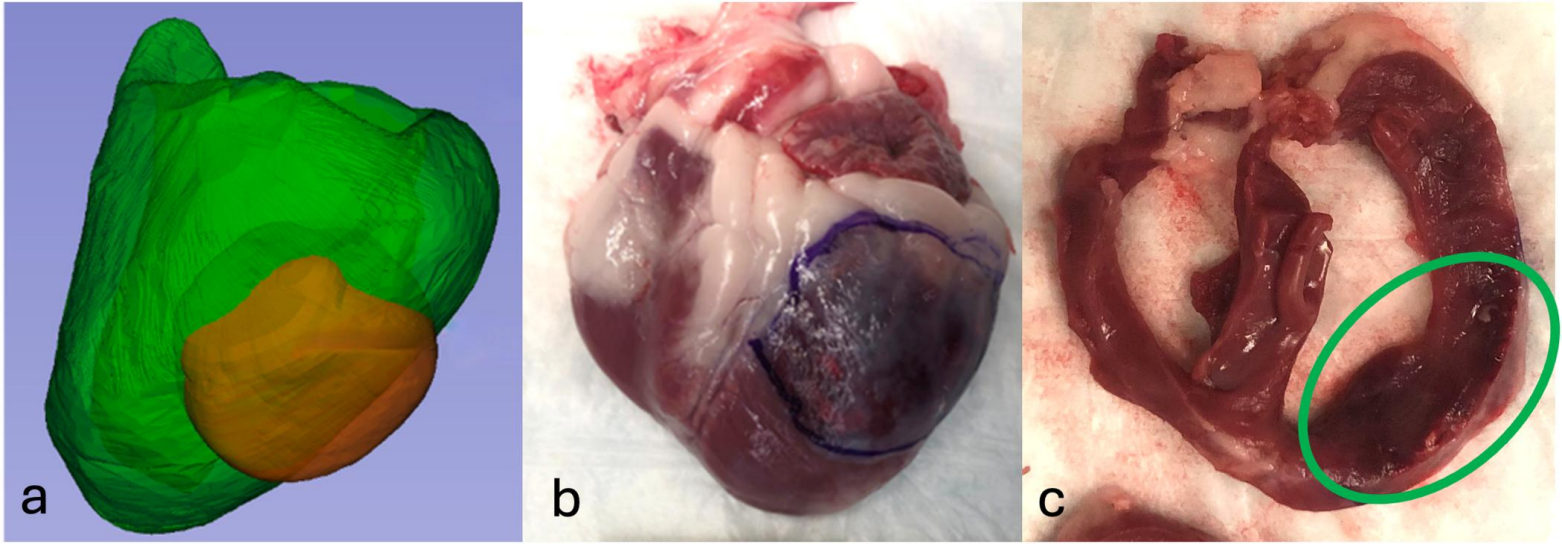
(a) Left ventricle (green) and radiation target (brown) from a dual‐gated treatment plan for one example animal; (b) surgical marker‐drawn lesion margins at necropsy; and (c) photograph of a heart slice at necropsy with transmural lesion (green area) on left ventricle free wall.

### 3.3 Correlation of Necropsy and Treatment Plan and Gating

Table 2 summarizes the visual lesion sizes and estimated volumes of the irradiated LVFWs receiving at least 95% of the prescription dose (i.e., IDV_95%). Macroscopic lesions were smaller in animals undergoing dual‐gated irradiation (21 ± 1 cm^3^, *N* = 2) compared to those undergoing single‐gated irradiation (31 ± 7 cm^3^, *N* = 2), although the difference was not statistically significant (*p* = 0.3). The measured transmural lesion volumes of dual‐gated animals (*N* = 2) matched the estimated IDV_95% by a median of 3%, whereas those of single‐gated animals (*N* = 2) were larger than expected by a median 77%. The differences between the groups were not significant (*p* = 0.3).

**Table 2:** Lesion size results.

	Protocol test		Analysis			
Animal identifier	1	2	3	4	5	6
Gating type	Dual	Single	Dual	Single	Dual	Single
Starting volume for transmural target delineation	3.5 cm diameter sphere	2.5 cm diameter sphere				
CardTV at baseline [cm^3^]	9.7	9.4	5.6	5.8	6.0	3.7
CardTV at termination [cm^3^]	16.1	20.9	9.8	11.1	12.1	7.9
Planning target volume at baseline [cm^3^]	37.0	43.0	21.0	24.0	21.0	20.0
IDV_95% [cm^3^]	36.1	33.1	17.7	16.7	22.4	18.1
Measured lesion size [cm^3^]	16.0	52.0	20.0	26.0	21.0	36.0

*Note:* The cardiac target volumes (CardTV) are shown at two time points (baseline and termination). IDV_95% represents the irradiated volume of LVFW which received at least 95% of the Rx dose (35 GyRBE).

## 4 Discussion

In the present study, we evaluated the safety and feasibility of single‐ and dual‐gated single‐fraction proton STAR using a large animal model and a clinically relevant workflow. The lesion outcomes of single‐ or dual‐gated scanned irradiations to a target in the LVFW were compared. We found that: (i) No significant systemic side effects or animal morbidity and mortality were observed following radiation. (ii) Dual‐gated irradiation was associated with a substantially decreased radiation exposure of myocardium outside of the target volume. (iii) Dual gating was associated with lesion sizes not far from those planned.

### 4.1 STAR

An increasing number of clinical centers are using STAR to treat refractory VT patients. Despite the diversity in imaging, planning, and delivery techniques, the first results show substantial reductions in VT burden and implantable cardioverter‐defibrillator shocks, and a low frequency of treatment‐related serious adverse events [7]. While the majority of patients have been treated with radiotherapy based on photons, proton‐based STAR is actively investigated: a first‐in‐human case was reported [21] and a more recent study on seven patients [22] showed that radiotherapy based on protons is also feasible, because it promises to significantly reduce radiation exposure to non‐target cardiac substructures and surrounding extracardiac OARs [23]. This study further explores the novel use of real‐time monitoring and cardiorespiratory gating to realize the full potential of proton‐based STAR.

### 4.2 Motion Mitigation

Mitigation techniques for respiratory motion, such as gating, are employed as a standard in cardiac imaging, cardiac electrophysiology, and radiotherapy for malignant diseases [24]. In the latter, gating has been shown to be associated with significantly better disease control and outcomes [25]. In STAR‐related motion studies, cardiac gating reduced the motion influence by approximately 50% in all directions and treatment area by up to 50% [26, 27]. In the presence of significant changes in target position/shape across the cardiac cycle, the use of cardiac‐gated proton delivery reduced the dose to healthy myocardium and OARs [23], which may translate into a lower risk of late cardiac or extracardiac toxicity.

The implementation of respiratory gating in the setting of STAR for VT was also shown to lead to a smaller PTV when compared to non‐gated volumes [28]. The implementation of dual‐gating compared with single‐gating resulted in a reduction in the average doses to OAR in patients undergoing irradiation therapy for cardiac sarcoma [29]. In another feasibility study of an ultrasound‐based gating device, three healthy swine were successfully irradiated with single‐ and dual‐gated proton radiotherapy, demonstrating accurate delivery of radiation to the target location and a significant decrease in the ITV when comparing dual‐ and non‐gated proton radiotherapy [11].

The present study provides the first data from a study with a clinically relevant workflow that shows that the application of dual gating enables the creation of lesions of a size reasonably close to the actual planned sizes (Graphical Abstract Image). If the results are reproducible in clinical use, this would allow for delivery of greater radiation doses to the target without increasing OAR doses; alternatively, reduce OAR exposure at currently used target doses. Such benefits remain conjectural at present, however, given that the optimal target doses for STAR have yet to be consistently defined.

When not using gating, the margin‐based approaches are based on cardiorespiratory motion during the pre‐imaging and treatment planning process, introducing an additional potential source of error. Recent multicenter collaborative work on retrospective metadata analysis and simulations has shown heterogeneity of motion among STAR targets and patients. Thus, accounting for cardiorespiratory motion optimizes STAR, leading to safer, potentially more effective radiation delivery, particularly when particle beams are used [6, 30].

### 4.3 ECG and Ultrasound‐Based Cardiorespiratory Gating for the Heart

No cardiorespiratory gating system is yet available for clinical use. In the present study, the workflow to image, plan, and deliver radiation therapy with the ECG and ultrasound‐based cardiorespiratory gating system for the heart was found to be feasible in all animals. The feasibility of using the system for imaging VT patients in STAR treatment position and to perform high‐quality proton and photon STAR treatment plans was demonstrated in previous publications [31, 32]. A recently published study by Rossi et al. shows that integrating CardioKit into the treatment planning workflow for STAR is feasible and requires only minor adjustments. However, there are currently no clinical studies published using dual‐gated radiation delivery. Ultrasound and ECG‐gated imaging are routinely employed in radiology and cardiology. This technology can also facilitate prolonged, real‐time, noninvasive imaging and localization of moving targets for STAR procedures across multiple radiation modalities. Additionally, it allows for direct imaging of the soft‐tissue target area, eliminating the need for surrogate markers. An alternative approach to cardiac gating based on MRI is currently being investigated in an experimental setup with an MRI‐linac [33].

### 4.4 Noninvasive Outcome Assessment of Myocardial Irradiation

In this study, [^18^F]‐FTPP^+^ PET‐CT was employed successfully to noninvasively monitor the cardiac tissue membrane potential in response to proton beam radiation and confirm radiation delivery to the intended target. The cardiac tissue membrane potential is a proxy of the mitochondrial membrane potential and an indicator of mitochondrial function. This noninvasive control method was essential for this study, since the treated subjects were healthy. An effect on the membrane potential was detectable in the expected areas at the 14‐week follow‐up. The differences in membrane potential compared to control areas were not statistically significant, likely due to the small sample size, but the effect was confirmed by necropsy. The study results suggest that this method could be used as an additional tool to assess effective delivery for STAR in the future. However, further studies are needed to confirm the validity of the method on infarcted hearts.

### 4.5 Impact of Dual Gating on Lesion Outcomes and Exposure of Myocardium to Irradiation

Macroscopic analysis showed a numerical difference in transmural lesion sizes between the single‐gated and dual‐gated animal groups, with smaller lesions in the dual‐gated group. Exposure of myocardium outside the CardTV was substantially lower with dual‐gated irradiation, and lesion sizes closer to those planned. The differences were not statistically significant, probably owing to the small sample size. A likely mechanism for why the observed lesion sizes were larger than expected for single‐gated animals is shown in Figure 8. Cardiac contraction pulls the surrounding healthy myocardium into the irradiation volume (i.e., IDV_95%) when the CardTV is radiated in the absence of cardiac gating. Our results confirm previous findings that showed that irradiated volumes are significantly reduced when using dual gating compared with no gating [11]. No significant systemic side effects or animal morbidity and mortality were observed following irradiation.

**Figure 8:**
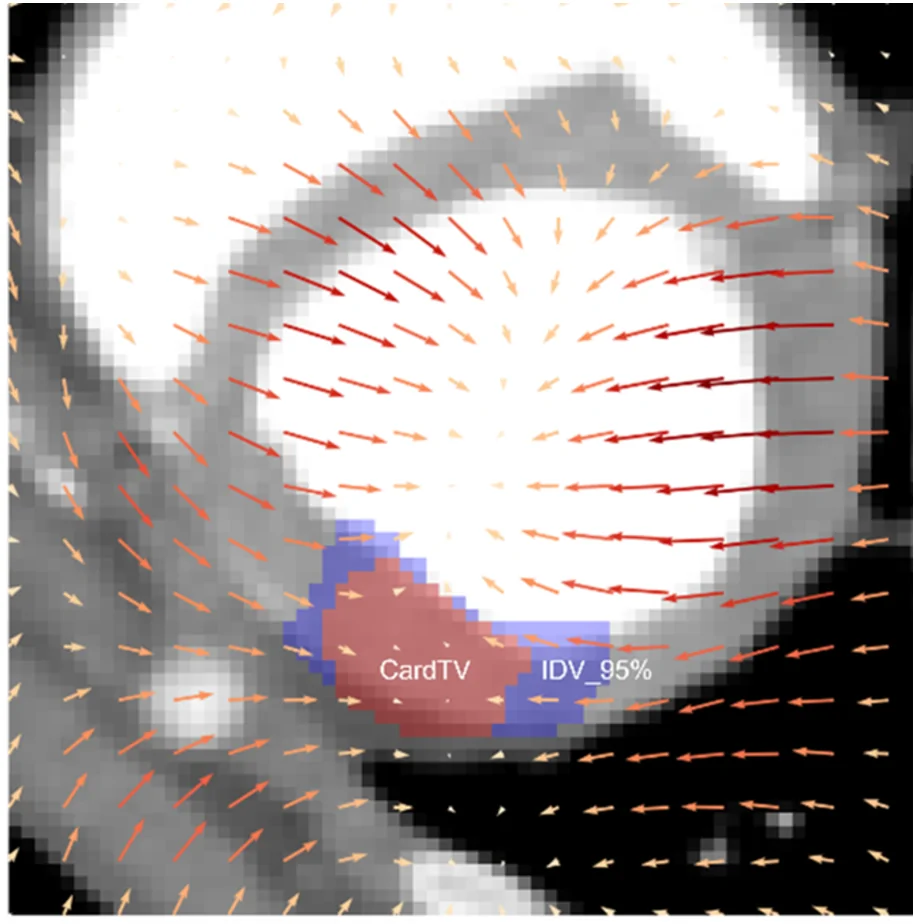
Illustration of tissue deformation in the form of arrows as the myocardium contracts from 80% cardiac phase to 40%. Cardiac contraction (direction and magnitude of motion symbolized by the deformation vectors) pulls the surrounding healthy myocardium into the irradiation volume (i.e., IDV_95%) when the CardTV is irradiated in the absence of cardiac gating.

In summary, our study found a reduction in treatment size expansion using cardio‐respiratory dual‐gating, while creating transmural lesions in almost the exact planned target area and dosimetrically less irradiation of non‐target tissues. Although ECG‐based gating is clinically used for cardiac imaging, no commercial solution is yet available for cardiac‐gated delivery of radiation therapy. With the newly developed CardioKit system, we were able to confirm that using a dual‐gating approach for VT radioablation has the potential to improve safety and, in turn, allow the use of higher doses of proton beams. These promising results need to be confirmed in human studies.

## 5 Limitations

This was a preclinical animal study with several limitations. The number of included animals was chosen to enable a workflow‐feasibility study and not for a formal comparison of dual‐ versus single gated radiation delivery, which limited the statistical power of this analysis. All imaging and treatment interventions were performed under general anesthesia and ventilation to immobilize the animals, whereas in the clinical setting, patients would be planned to undergo treatment, free‐breathing, and awake, leading to more variability in target movement. In addition, our animals were healthy subjects without myocardial scar. On the other hand, the use of a porcine model resulted in considerably higher heart rates and poorer transthoracic echocardiography windows compared to a VT patient population, making the approach more challenging than in humans. The first animal showed only an epicardial lesion, which is likely due to sub‐par alignment prior to treatment (i.e., poor concordance of treatment plan and delivery on the treatment day)—an issue which was corrected for subsequent animals. Some PET data sets were excluded from the analysis due to insufficient image motion necessary for correction (one dual‐gated animal). The PET‐CT membrane‐potential analysis was not blinded. Future studies are necessary to confirm the safety and feasibility of this method in human hearts. Importantly, as cardiorespiratory motion has been reported to be site‐specific [7], the impact of clinical application of motion mitigation will likely differ for different target locations.

## 6 Conclusions

Both single and dual‐gated proton single‐fraction delivery with a respiratory or cardiorespiratory gating device was found to be clinically feasible and safe for all animals. Dual‐gated irradiation was associated with lower exposure of myocardium outside the target volume to radiation while more accurately achieving the planned lesion size. Future studies are necessary to confirm the safety and feasibility of cardio‐respiratory gating in human VT patients.

The study was co‐sponsored by a collaborative research agreement with EBAMed SA.

## Conflicts of Interest

Dr H. Immo Lehmann has provided consulting services to EBAMed and received research funding from Philips. Adriano Garonna is an employee of EBAMed. The other authors declare no conflicts of interest.

## Data Availability

The data that support the findings of this study are available on request from the corresponding author. The data are not publicly available due to privacy or ethical restrictions.
